# Revision of fossil species of *Deinodryinus*, with description of a new species (Hymenoptera, Dryinidae)

**DOI:** 10.3897/zookeys.130.1326

**Published:** 2011-09-24

**Authors:** Adalgisa Guglielmino, Massimo Olmi

**Affiliations:** 1 Department of Plant Protection, University of Tuscia, Via S. Camillo de Lellis, I-01100 Viterbo, Italy

**Keywords:** Taxonomy, Deinodryinus velteni, Baltic amber, key, Dryinidae, Anteoninae

## Abstract

*Deinodryinus velteni* **sp. n.** is described from middle Eocene Baltic amber. The species differs from other fossil Palaearctic species of *Deinodryinus* Perkins owing to the shape of the antenna (clavate, with distal part very thickened), the large compound eyes, and the distal part of the stigmal vein much longer than the proximal part. A revision and a key to the fossil Palaearctic species of *Deinodryinus* Perkins, 1907 is presented.

## Introduction

Dryinidae (Hymenoptera, Chrysidoidea) are parasitoids of Auchenorrhyncha (Guglielmino and Olmi 1997, 2006, 2007). The genus *Deinodryinus* Perkins, 1907 is present in all zoogeographical regions and a member of the subfamily Anteoninae. One hundred and forty-eight species of *Deinodryinus* have been described from throughout the world, of which only two are fossil species (Olmi 1984, 1999; Olmi et al. 2010; Ponomarenko 1975): *Deinodryinus areolatus* (Ponomarenko, 1975), from Baltic amber, and *Deinodryinus? aptianus* Olmi, Rasnitsyn & Guglielmino, 2010, a compression fossil from Early Cretaceous marl of the Khurilt rock unit (Mongolia). The latter species is tentatively placed within *Deinodryinus* given that it is uncertain whether the attribution of this Early Cretaceous species to a modern genus is justified. Insufficient characters were preserved in *Deinodryinus? aptianus* to support its placement in a new generic taxon.

Recently we have discovered an additional new fossil species of *Deinodryinus* from Baltic amber, and the taxon is described herein.

## Material and methods

The descriptions follow the terminology used by (Olmi (1984, 1994a, 1999). The measurements reported are relative except for the total length (head to abdominal tip, without the antennae) and the length of some parts of the body, which are expressed in millimetres.

The redescriptions of *Deinodryinus? aptianus* and *Deinodryinus areolatus,* respectively by Olmi et al. (2010) and Ponomarenko (1975), are provided for the sake of completeness. Information on the fossil deposits under consideration are provided by Rasnitsyn and Quicke (2002).

The material studied in the present paper is deposited in the following institutions:

**PIN** A.A. Borissiak Palaeontological Institute,Russian Academy of Sciences, Moscow (Russia).

**SNMS** Staatliches Museum für Naturkunde Stuttgart, Abt. Paläontologie–Sektion Bernstein, Stuttgart (Germany).

## Systematic paleontology

### Genus Deinodryinus

Perkins, 1907

http://species-id.net/wiki/Deinodryinus

Deinodryinus Perkins, 1907: 45. Type species: *Deinodryinus paradoxus* Perkins, 1907, designated by Muesebeck and Walkley 1951.
Trisanteon Kieffer, 1913: 300 (synonymized by Olmi 1984); type species: *Trisanteon hirticornis* (Kieffer, 1911), monotypic and original designation.
Electrodryinus Ponomarenko, 1975: 126 (synonymized by Olmi 1984); type species: *Electrodryinus areolatus* Ponomarenko, 1975, monotypic.
Prioranteon Olmi, 1984: 589 (synonymized by Olmi 2007); type species: *Prioranteon casalei* Olmi, 1984, original designation.

#### Diagnosis.

Female: macropterous or micropterous; palpal formula 6/3; in macropterous specimens forewing usually with distal part of stigmal vein longer than proximal part, less frequently as long as, or shorter than proximal part; occipital carina complete; vertex frequently with two strong oblique keels connecting posterior ocelli to occipital carina; pronotum with distinct anterior collar and posterior disc; foreleg chelate; enlarged claw with inner proximal prominence not bearing bristles, with 1–2 bristles or peg-like hairs located further distally than proximal prominence; tibial spurs 1/1/2. Male: always macropterous (even if female micropterous); palpal formula 6/3; forewing usually with distal part of stigmal vein longer than proximal part, less frequently as long as, or shorter than proximal part; forewing usually with pterostigma four or more than four times as long as broad; antennal hairs usually much longer than breadth of segments, less frequently shorter than breadth of segments; vertex frequently with two strong oblique keels connecting posterior ocelli to occipital carina; paramere without dorsal process, usually with more or less large inner branch wrapping penis, less frequently with reduced inner branch; tibial spurs 1/1/2.

#### Distribution.

Worldwide.

#### Hosts.

Cicadellidae (Guglielmino and Olmi 2007).

#### Species.

Presently with 152 living and fossil species.

#### Key to the fossil species of *Deinodryinus*

Females

**Table:** 

1	Antenna filiform (Fig. 1); compound eye shorter than one-half length of head (Fig. 1)	*Deinodryinus aptianus* Olmi, Rasnitsyn & Guglielmino
–	Antenna clavate (Figs 2, 3); compound eye longer than one-half length of head (Figs 2, 3)	2
2	Distal part of stigmal vein much longer than proximal part (Fig. 3)	*Deinodryinus velteni* sp. n.
–	Distal part of stigmal vein about as long as proximal part (Fig. 2)	*Deinodryinus areolatus* (Ponomarenko)

Males: Unknown.

### Deinodryinus? aptianus

Olmi, Rasnitsyn & Guglielmino

Fig. 1

Deinodryinus? aptianus Olmi, Rasnitsyn & Guglielmino 2010: 30.

#### Material examined.

**Type**: *Holotype*, female, MONGOLIA: Central Mongolia, Bayanhongor Aimag, 5–8 km N Bon Tsagan Nuur Lake, outcrop 87, bed 8, impressed in marl of the Khurilt rock unit probably of Aptian age (Early Cretaceous) (100–115 mybp)(PIN, No. 3559/4586).

#### Diagnosis.

**Figure 1.**
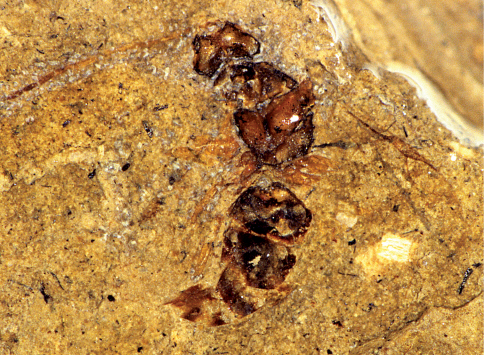
*Deinodryinus? aptianus*. Female holotype (from Olmi et al. 2010). Length 6.2 mm.

Female with antenna filiform and compound eye small (Fig. 1).

#### Redescription.

*Female*: macropterous; length 6.2 mm; length of main regions: head: 0.87 mm; antennae: 3.06 mm; mesosoma: 1.37 mm; prothorax: 0.87 mm; mesothorax + metathorax + propodeum: 1.37 mm; metasoma: 3.12 mm. Antenna filiform (Fig. 1); antennal segments in following proportions: 14:12:14:12:13:17:15:13:12:15; length/breadth ratio of antennal segments 8–10: 8^th^: 13:3; 9^th^: 12:3; 10^th^: 15:3. Head only visible from ventral side. Occiput very deeply excavated, with hipostomal bridge short (in ventral side, length of occiput: 0.62 mm; hipostomal bridge: 0.62 mm; oral fossa: 0.43 mm). Palpi not visible. Compound eye small (Fig. 1). Propleura normal (as in extant dryinids). Profemur very large, covering ventral side of mesothorax. Forewing hyaline, with three basal cells completely enclosed by pigmented veins. Marginal cell closed. Stigmal vein regularly curved, distal part much longer than proximal part. Pterostigma very narrow, with following length/breadth ratio: 35:7. Petiole very short. Ovipositor present. Legs only partly visible. Profemur very large (length/breadth ratio: 39:18), as in extant chelate female of dryinids. Chela present, hardly visible. Remainder of forelegs, partly missing. Mid- and hindlegs partly missing. Length of mesocoxa: 0.62 mm. Length of metacoxa: 0.81 mm. Tibial spurs not visible.

*Male*: unknown.

#### Hosts.

Unknown.

#### Remarks.

This fossil is only visible in ventral aspect and is difficult accordingly to place within a particular genus. In addition, the legs are partly missing and the chela is hardly visible. However, it is possible to identify tentatively this specimen as a species of the extant genus *Deinodryinus* given the shape of the pterostigma and stigmal vein and for the presence in the forewing of three basal cells completely enclosed by pigmented veins. Among Anteoninae, the above characters may also place this specimen in *Lonchodryinus* Kieffer, 1905, but *Lonchodryinus* has the hypostomal bridge much shorter than in *Deinodryinus*. Given that the fossil exhibits the condition in the latter genus the authors placed the species in *Deinodryinus*. Among Dryinidae, the above characters of the forewing may also place this specimen in Dryininae and Gonatopodinae but because the occiput is less excavated in Dryininae and Gonatopodinae than in *Deinodryinus*, and in *Deinodryinus? aptianus*, attribution to *Deinodryinus* is more justified. Another unusual character of *Deinodryinus? aptianus* is the shape of the antennae: they are filiform, as in males of Dryinidae, whereas in females usually they are clavate (Olmi et al. 2010).

### Deinodryinus areolatus

(N. Ponomarenko)

http://species-id.net/wiki/Deinodryinus_areolatus

Fig. 2

Electrodryinus areolatus N. Ponomarenko 1975: 128.
Deinodryinus areolatus (N. Ponomarenko): Olmi 1984: 121.
Deinodryinus areolatus (N. Ponomarenko): Olmi 1995: 268.
Deinodryinus areolatus (N. Ponomarenko): Olmi and Bechly 2001: 41.

#### Material examined.

**Type**: *Holotype*, female, Eocene Baltic amber (40–45 mybp)(PIN, No. 964/60).

#### Diagnosis.

**Figure 2.**
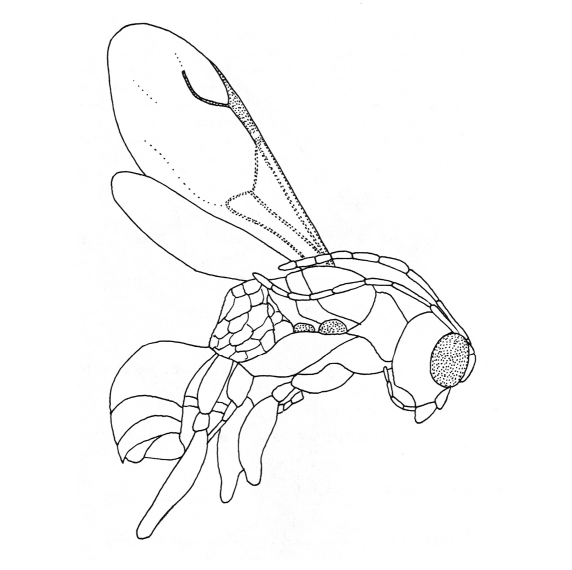
*Deinodryinus areolatus*. Female holotype (from Olmi 1984). Length 4.5 mm.

Female with antenna clavate and compound eye large (Fig. 2); distal part of stigmal vein about as long as proximal part (Fig. 2).

#### Redescription.

*Female*: macropterous; length 4.5 mm. Head black, except anterior region of face brown; clypeus testaceous, except central brown spot; mandible testaceous, except teeth and proximal region brown; antenna testaceous; mesosoma and metasoma black; legs brown-testaceous. Antenna 10-segmented, clavate, densely hairy, less than three times as long as head (157:63); antennal segments in following proportions: 17:10:30:22:18:15:11:11:10:13. Clypeus with anterior margin weakly emarginated. Antennal torulus distinctly separated from epistomal sulcus. Mandible with four teeth progressing larger from anterior one to posterior. Compound eye apparently bare, normally protruding. Subocular sulcus present. Occipital carina complete. Temple prominent. Posterior ocelli hardly visible, not touching occipital carina. Palpal formula 6/3. Pronotum not crossed by transverse impressions; pronotal tubercle reaching tegula; posterior margin of pronotum longer than anterior margin. Thoracic structure similar to that of extant *Deinodryinus*. Scutum shiny, finely punctate, longer than pronotum (20:15). Notauli complete, posteriorly separated. Propodeum reticulate rugose, with areolae very broad; dorsal surface approximately as long as posterior surface; posterior surface very steep, not distincly visible. Petiole distinct. Forewing hyaline, without dark transverse bands, with normal venation of Anteoninae; pterostigma narrow, more than four times as long as broad (40:8); marginal cell open; distal part of stigmal vein about as long as proximal part (18:17); stigmal vein not S-shaped. Forewing with usual three basal cells clearly enclosed by pigmented veins (costal, median, and submedian cells). Shape of wings usual for *Deinodryinus*. Protrochanter not slender, without proximal slender stalk, slightly longer than broad (10:6). Segment 3 of protarsus produced into hook; segment 1 of protarsus slightly shorter than segment 4 (15:17). Forelegs chelate. Chela without rudimentary claw. Arolium much shorter than enlarged claw (7:32). Enlarged claw without subapical tooth and other teeth. Segment 5 of protarsus about as long as enlarged claw, with lamellae hardly visible. Tibial spurs 1/1/2.

*Male*: unknown.

#### Hosts.

Unknown.

#### Remarks.

In the holotype the sculpture of the vertex, face, and pronotum is hardly visible; the scutellum and metanotum are not visible.

### Deinodryinus velteni

Guglielmino & Olmi sp. n.

urn:lsid:zoobank.org:act:AD16D59C-D1EF-4DF9-8ACC-F1E55C42DAA2

http://species-id.net/wiki/Deinodryinus_velteni

Fig. 3

#### Etymology.

The species is named after Mr. Jürgen Velten (Idstein, Germany).

#### Material examined.

**Type**: *Holotype*, female, Eocene Baltic amber (40–45 mybp) (SMSN).

#### Diagnosis.

**Figure 3.**
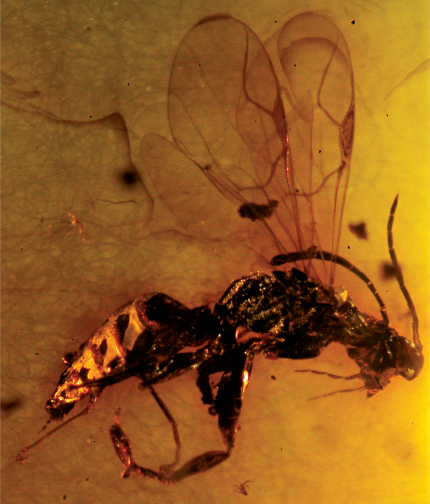
*Deinodryinus velteni*. Female holotype. Length 4.0 mm.

Female with antenna clavate and compound eye large (Fig. 3); distal part of stigmal vein much longer than proximal part (Fig. 3).

#### Description.

*Female*: macropterous; length 4.0 mm. Colour apparently brown-black, except palpi testaceous. Antenna 10-segmented, clavate, short, covered with dense and short hairs, thickened distally; antennal rhinaria absent; antennal segments in following proportions: 5:7:8:13:10:9:8:6:6:9; antenna much shorter than body, approximately three times as long as head (head length dorsally measured from occipital carina behind ocelli to distal apex of mandible): 75:25. Head only partly visible, slightly convex, dull, apparently granulated and hairless; occiput excavated; compound eye normally bulging; ocelli partly visible; ocellar triangle apparently equilateral; temple distinct. Pronotum long, crossed by anterior strong transverse impression, with posterior disc, without posterior collar; pronotum apparently almost glabrous, shiny, slightly shorter than head (22:25); pronotal disc flat posteriorly, much longer than anterior collar; pronotal tubercle reaching tegula. Scutum dull, apparently glabrous, granulated, slightly shorter than pronotum (19:22). Notauli complete, posteriorly separated; minimum distance between notauli approximately as long as antennal segment 2. Scutellum very humped (Fig. 3), much shorter than scutum (8:19). Metanotum very humped (Fig. 3), shorter than scutellum (6:8). Propodeum longer than scutum (29:19), apparently reticulate rugose; sculpture of dorsal and posterior surfaces visible only laterally. Metapleura dull, rugose and partly sculptured by transverse keels. Epicnemium present. Shape of head, scutum, scutellum, metanotum and propodeum usual for Anteoninae. Forewing apparently completely weakly darkened, with usual venation of Anteoninae. Pterostigma long and narrow, much longer than broad (30:6). Pterostigma shape similar to that of extant *Deinodryinus*. Marginal cell open. Stigmal vein not S-shaped, with distal part much longer than proximal part (20:14); stigmal vein forming an angle between proximal and distal parts. Forewing with usual three basal cells clearly enclosed by pigmented veins (costal, median and submedian cells). Hindwing apparently slightly darkened. Hindwing shape usual for Anteoninae. Foreleg segments in following proportions: 30 (coxa): 7 (trochanter): 43 (femur): 29 (tibia): 7 (tarsomere 1): 3 (tarsomere 2): 5 (tarsomere 3): 11 (tarsomere 4): 24 (tarsomere 5). Foreleg chelate. Enlarged claw slightly shorter than tarsomere 5 (22:24). Protrochanter short, slightly longer than broad (7:5). Protrochanter shape similar to that of Anteoninae. Tarsomeres 2 and 3 of protarsus produced into a hook. Rudimentary claw absent. Arolium much shorter than enlarged claw (8:22). Distal apex of enlarged claw apparently pointed. Tarsomere 5 of protarsus with numerous lamellae on inner margin and distal apex. Midleg segments in following proportions: 12 (coxa): 8 (trochanter): 26 (femur): 24 (tibia): 22 (tarsomere 1): 9 (tarsomere 2): 6 (tarsomere 3): 4 (tarsomere 4): 6 (tarsomere 5). Hindleg segments in following proportions: 19 (coxa): 5 (trochanter): 33 (femur): 32 (tibia): 23 (tarsomere 1): 10 (tarsomere 2): 8 (tarsomere 3): 4 (tarsomere 4): 6 (tarsomere 5). Petiole shape and length usual for Anteoninae. Palpal formula 6/3. Shape, length and breadth of wings usual for Anteoninae. Shape and morphology of body usual for Anteoninae. Tibial spurs 1/1/2.

*Male*: unknown.

#### Hosts.

Unknown.

#### Remarks.

In the holotype the clypeus, mandibular teeth, frontal line, occipital carina, POL, OL, OOL, OPL, TL, and posterior surface of the propodeum are not visible; the sculpture of the pronotum, scutellum, metanotum, and mesopleura is not distinct; the enlarged claw is only partly visible because of a closed chela so that it is not possible to see if there are subapical teeth and lamellae; and tarsomere 5 of the protarsus is only partly visible so that it is impossible to count the lamellae and to see if there are one or two rows of lamellae.

## Discussion

With 152 species, the genus *Deinodryinus* is present in all zoogeographical regions: six species are Palaearctic; 22 Afrotropical; nine Oriental; three Nearctic; 110 Neotropical; and two Australian. The only known fossil species have been found in the Palaearctic region, and all have been summarized in the present paper. Because of its geographic distribution, *Deinodryinus* is considered a ‘Pangean’ genus (Olmi 1994b). The genus perhaps originated in Central and South America, where the greatest number of extant species are present (110), although centres of origin do not always reside in areas today harboring the greatest species diversity and so this hypothesis requires phylogenetic testing. Almost all species live in tropical or subtropical countries. Very few are the species living in temperate countries: two in the Palaearctic region [*Deinodryinus biroi* (Olmi, 1984) and *Deinodryinus hispanicus* (Olmi, 1991)], and one in the Nearctic region [*Deinodryinus atriventris* (Cresson, 1872)]. The presence of fossil species in Baltic amber and Early Cretaceous Mongolia marl perhaps indicates that these areas had relatively warm climates in the past, conclusions supported by other faunal and floral elements in these deposits, and that the genus had a much wider distribution.

From a morphological standpoint, *Deinodryinus areolatus* and *Deinodryinus velteni* do not exhibit significant differences from extant species of the genus. This is common with many fossil dryinids. By contrast, *Deinodryinus? aptianus*, visible only in ventral aspect, with legs partly missing and the chela hardly distinct, is difficult to interpret. As mentioned above, attribution to *Deinodryinus* is only tentative and based on the shape of the pterostigma and stigmal vein and by the presence in the forewing of three basal cells completely enclosed by pigmented veins. However, the presence of filiform antennae, a character present in very few females of dryinids and rare in *Deinodryinus* (only the females of *Deinodryinus benoiti* Olmi, 1984, from Madagascar, and *Deinodryinus colombianus* Olmi, 1984, from South America, have filiform antennae), makes this attribution somewhat suspect. Accordingly, our assignment of this species to *Deinodryinus* remains speculative and we hope for the eventual discovery of more completely preserved material so as to clarify the generic status of this ancient taxon.

## Supplementary Material

XML Treatment for Deinodryinus

XML Treatment for Deinodryinus? aptianus

XML Treatment for Deinodryinus areolatus

XML Treatment for Deinodryinus velteni
